# Optical Coherence Tomography: Potential Clinical Applications

**DOI:** 10.1007/s12410-012-9140-x

**Published:** 2012-05-03

**Authors:** Antonios Karanasos, Jurgen Ligthart, Karen Witberg, Gijs van Soest, Nico Bruining, Evelyn Regar

**Affiliations:** Department of Cardiology, Erasmus University Medical Center, Thoraxcenter, BA-585, ‘s Gravendijkwal 230, 3015 CE Rotterdam, The Netherlands

**Keywords:** Optical coherence tomography, Intravascular imaging, Percutaneous coronary intervention, Stent coverage, Restenosis, Stent thrombosis, Fractional flow reserve, Intravascular ultrasound, Procedural guidance, Coronary angiography, Stent, Plaque, Atheromatosis

## Abstract

Optical coherence tomography (OCT) is a novel intravascular imaging modality using near-infrared light. By OCT it is possible to obtain high-resolution cross-sectional images of the vascular wall structure and assess the acute and long-term effects of percutaneous coronary intervention. For the time being OCT has been mainly used in research providing new insights into the pathophysiology of the atheromatic plaque and of the vascular response to stenting, however, it seems that there is potential for clinical application of OCT in various fields, such as pre-interventional evaluation of coronary arteries, procedural guidance in coronary interventions, and follow-up assessment of vascular healing after stent implantation. This review will focus on the potential and advantages of OCT in the clinical practice of a catheterization laboratory.

## Introduction

Since the introduction of percutaneous coronary intervention (PCI), coronary angiography has been the tool for assessing the need for revascularization, as well as for assessing the results of the interventions. The advent of intravascular ultrasound (IVUS) has opened a new chapter in PCI guidance, enabling the transition from assessment of the lumen to the direct assessment of plaque and vessel morphology and the evaluation of the vessel wall response to stenting. Utilizing this approach, IVUS studies have provided useful insights into the pathogenesis and prevention of stent failure [1–3] as well as the dynamic nature of atherosclerosis and the impact of medical therapy [4–6]. Despite, however, the enormous contribution of IVUS, it is limited by low axial resolution (100–150 μm) and poor capability to discriminate different plaque components that precludes assessment of specific plaque and stent characteristics. Technological advances have led to the development of new intravascular imaging methods. We will discuss the potential and advantages of optical coherence tomography (OCT) in the clinical practice of a catheterization laboratory. Table 1 summarizes some potential clinical applications of OCT.

**Table 1:** Potential clinical applications of optical coherence tomography (OCT)

Setting	Application
Lesion evaluation	Assessment of culprit lesion in acute coronary syndromes: evaluation for plaque rupture and/or thrombus in patients without angiographically evident culprit lesion
	Evaluation of lesions with angiographic haziness: differential diagnosis between thrombus, dissection, heavy calcification
	Determination about presence or absence of plaque (e.g. in coronary spasm)
Pre-procedural assessment	Luminal measurements for selection of balloon and stent dimensions
	Assessment of plaque morphology in order to guide therapeutic strategy and device selection (rotablation, cutting balloon, etc.)
	Evaluation of the optimal location in the vessel for implantation of a coronary stent
	Use for tracking the exact guidewire position (i.e. in chronic total occlusion or in bifurcation stenting)
	Use in bifurcation intervention (assessment of carina, ostia of side-branches, stent cell geometry)
Post-procedural assessment	Assessment of stent expansion (detection of under-expansion, residual stenosis, incomplete stent apposition)
	Assessment of vascular injury: detection of edge dissections, tissue protrusion, intra-stent thrombus
	Assessment of intervention by adjunctive devices: measurement of luminal enlargement after cutting balloon angioplasty, assessment of the reduction of calcification after rotablation
	Assessment of adjunctive therapies in acute coronary syndromes: evaluation of residual thrombus burden after thrombectomy or selective administration of IIb/IIIa antagonists
Follow-up stent assessment	Mid-term and long-term assessment of stent safety and efficacy: evaluation of stent restenosis (quantitative and qualitative), stent thrombosis, and stent coverage as a surrogate for vessel healing
	Monitoring of the bioresorption and the healing response after implantation of bioresorbable scaffolds

## Basic Principles of OCT Imaging

OCT is an intravascular imaging method capable of proving cross-sectional images of the vascular wall with a high axial resolution (5–15 μm) and a lateral resolution of approximately 25 μm. This high resolution, however, comes at a cost of reduced penetration depth caused by the rapid attenuation of light waves in the tissue, and of the need to temporarily displace blood during imaging, because of the high scattering of light caused by erythrocytes. OCT in contrast to IVUS is based on emission of near-infrared light rather than acoustic waves for imaging. These infrared light waves reflect off the vascular wall and are then received and processed by the OCT imaging system. As the wavelength of light waves is far greater than those of the intravascular ultrasound, OCT cannot directly interpret the signal, but uses instead a technique called low-coherence interferometry in order to process the reflected signal. By this technique, an interferogram is created that can be analyzed by the imaging system in order to interpret the reflection as temporal delay or as tissue length (A-line) [7••].

### Time-Domain and Fourrier-Domain OCT Systems

There are two processing methods that are being used for intravascular OCT imaging: Time Domain OCT (TD-OCT), and the recently introduced Fourier Domain OCT (FD-OCT), which is also known as second generation OCT, Frequency Domain OCT, or Optical Frequency Domain Imaging (OFDI). FD-OCT systems can record A-lines at a very high speed, while improving noise-signal ratios resulting in greater penetration depth, without loss of vital detail or resolution. In this way, imaging acquisition time and contrast used is reduced, while longer pullbacks are acquired [8].

### Intravascular FD-OCT Systems and Image Acquisition Technique

Currently available FD-OCT systems are utilizing a short rapid-exchange monorail catheter for mounting of the optical probe. The optical signal is transmitted by a single-mode fiber, automatically mounted on the catheter, the focus of which is approximately 1 mm outside the catheter. In order to scan the vessel lengthwise, the catheter imaging tip is pulled back while rotating, inside a transparent sheath, allowing for retrieval of consecutive cross-sectional images for a length of 50–70 mm from the coronary artery. Both rotary and pullback motion are driven proximally by a motor outside the patient. For image acquisition, the FD-OCT catheter is initially advanced with the aid of a conventional guidewire distally to the desired segment, while the catheter position is indicated by radio-opaque markers. In order to create a blood-free environment during the acquisition, contrast warmed to 37 °C is injected, either manually, or by a power injector at a rate of 3–4 mL/sec. Pullback of the optical fiber is being performed simultaneously with the contrast injection.

### Safety and Efficacy of OCT Imaging

The first experimental and human studies of TD-OCT have shown the safety and feasibility of OCT imaging using a proximal occlusion balloon for blood clearance [9]. An alternative technique eliminating the need for proximal occlusion has also demonstrated a favorable safety profile [10]. In the first systematic attempt from a multicenter registry of 468 patients undergoing TD-OCT imaging either with the occlusive or the non-occlusive technique, to record the safety of optical coherence tomography, use of the non-occlusive technique was associated with a reduction in the rate of transient complications during image acquisition such as chest pain and ECG changes [11••]. The introduction of FD-OCT led to a significant improvement in the rate of images of usable quality by simultaneously reducing acquisition time and the rate of transient chest pain and ECG changes [8]. Furthermore, use of a FD-OCT system for guidance of intervention was associated with a favorable safety profile [12].

### Plaque Characterization and Quantitative Measurements

**Fig. 1:**
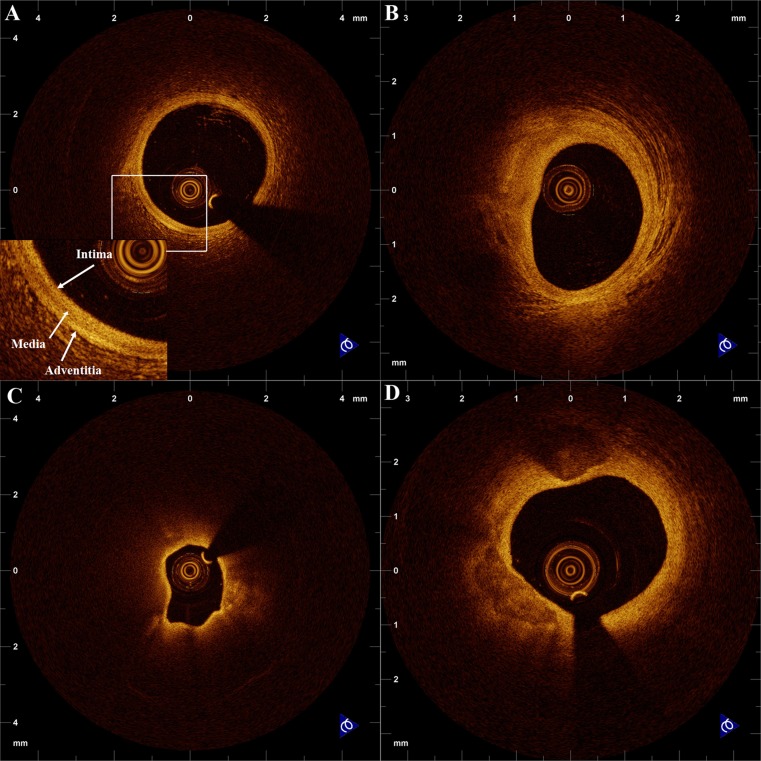
Representative optical coherence tomography (OCT) cross-sectional images of **a** normal vessel with high-power view demonstrating the three layers of the vascular wall: **b** fibrous plaque; **c** lipid-rich plaque; **d** fibrocalcific plaque

OCT is an imaging method that can accurately measure lumen and stent dimensions with high agreement with histological specimens and characterize tissue with high sensitivity and specificity. By OCT, it is possible to visualize all three arterial layers in a normal coronary artery, in accordance with pathological specimens [13]. OCT allows differentiation of fibrous, fibrocalcific, and lipid-rich tissue by visual assessment of the standard intensity image [14]. Briefly, fibrous plaque is seen as a high-intensity, low-attenuation area, lipid as a low-intensity, high-attenuation area with diffuse borders, while calcium as low-intensity, low-attenuation area with sharp borders (Fig. 1) [14]. OCT can also detect thrombus with very high sensitivity and specificity, and allows for distinction between red (erythrocyte-rich) and white (platelet-rich) thrombus [15].

Several studies have validated the ability of OCT to perform quantitative measurements of the lumen and stent area [7••, 16, 17]. Meanwhile, in vivo studies have confirmed that there is high correlation between OCT and IVUS measurements of vessel and stent dimensions, as well as that there is very low intra-observer and inter-observer variability in the assessment of stent and vessel area [18]. Measurements acquired by FD-OCT systems seem to be equally accurate and reproducible [19]. The recent introduction of algorithms allowing for the automated quantitative assessment of lumen dimensions enables the acceleration of the analysis process [20–22]. Various algorithms and software packages have been suggested for this approach [20], while the ability of these automatic quantification programs to detect with high correlation with histology and manual interpretation the luminal and stent borders allows for the rapid online assessment of the degree of stenosis, reference site diameter, and degree of neointimal hyperplasia [21, 22].

## Role of OCT in Pre-interventional Decision Making

### Intermediate Lesions

Accurate measurements of lumen area by OCT can be used for the evaluation of intermediate lesions. It has been shown in IVUS studies, that angiography often underestimates the plaque burden and the severity of stenosis [23]. Thus, evaluation of intermediate lesions has been a focus of invasive imaging. Functional assessment of intermediate lesions using fractional flow reserve (FFR) in patients with stable disease has been shown in large-scale studies to be linked with improved outcomes as it overcomes limitations of angiography [24]. Despite the existence of IVUS studies assessing quantitative indices of stenosis as a marker of the functional significance of a lesion, it remains questionable whether functional measurements can be replaced by anatomic measurements by IVUS or OCT. Taking into account, however, that various parameters, such as lesion length, lesion location, and plaque burden are factors also affecting the functional significance of a lesion, it is doubtful that the physiological significance of a lesion can be accurately assessed by measurement of minimal lumen area alone [25]. Moreover, a recent evaluation of IVUS and OCT to assess the physiological significance of a stenosis revealed that despite OCT measurements of minimal lumen area having greater diagnostic potential than IVUS for the assessment of the physiological significance of intermediate stenosis, anatomic lesion assessment by either modality correlates poorly to physiologic measurements performed by FFR [26]. Consequently, the current golden standard to assess hemodynamic relevance in a patient with stable angina is functional measurement by FFR (what to treat?), while OCT is helpful in guiding the procedure (how to treat?) or identification of the culprit and thrombosed lesion in acute coronary syndromes with intermediate lesions.

### Angiographic Haziness

**Fig. 2:**
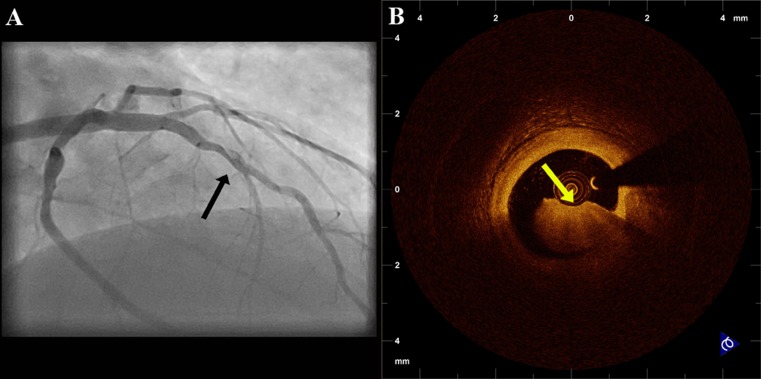
Evaluation by OCT of a patient with acute coronary syndrome and haziness in the coronary angiogram. OCT revealed the presence of thrombus

A field were OCT could substantially improve preprocedural assessment is in the evaluation of hazy lesions (Fig. 2). It is known that angiographic haziness could be due to a variety of morphological features such as thrombus, heavy calcification, extreme tortuosity, and intimal dissection. There have been several reports of utilizing OCT for the assessment of the nature of haziness, that have ranged from extreme vessel tortuosity to vessel dissection and recanalised thrombus [27–29]. Furthermore, the use of IVUS would be less helpful in such cases, as its diagnostic accuracy for complicated plaque and thrombus detection is limited [30].

### Evaluation of Coronary Spasm

Optical coherence tomography can also aid decision making in cases of acute coronary syndromes in patients where coronary spasm appears to be the culprit. Use of OCT in this subset of patients can exclude the presence of culprit atheromatic plaques and/or thrombus. The association of plaque morphology with coronary spasm has been evaluated by OCT, showing that the main underlying morphology is transient intima-media thickening [31]. Multiple lumen irregularities observed during the episode consisting of intimal bumps and intimal gathering disappear after intracoronary nitrate administration, giving place to normal vessel morphology [31].

### Assessment of Plaque Morphology

One of the advantages of OCT is that it can assist in the preprocedural evaluation of coronary lesions by providing information about the morphology of the lesions. A small study recently evaluated the complementary use of FD-OCT with FFR in order to assess angiographically ambiguous lesions. In this study, FFR was used in order to provide information about the functional significance of these lesions, while FD-OCT was used in order to assess the presence of unstable plaques and guide decision making in cases of ACS and FFR values >0.80 [32]. Moreover, the underlying plaque type has been associated with the outcome of the intervention. The presence of lipid-rich plaques by OCT has been associated with greater incidence of non-reflow and greater rise in biomarkers of cardiac necrosis in several studies [33]. Additionally, patients with unstable angina compared to stable patients had greater rates of acute malapposition and tissue protrusion post PCI, while they had higher rates of malapposition and incomplete coverage at 9-month follow-up, implying an association between complicated plaque morphology—which was more common in the unstable angina group—with impaired vessel healing after stenting [34].

It remains to be proven whether qualitative assessment of non-culprit lesions can guide decision for treatment and improve patient-related outcomes. A recent intravascular ultrasound study suggested an association of the presence of a non-culprit lesion with small lumen area, large plaque burden and plaque morphology, as identified by radiofrequency ultrasound analysis, with subsequent re-hospitalizations and events [6]. However, the prognostic value of intravascular ultrasound with radiofrequency signal analysis for vulnerable plaque identification and risk stratification still needs to be established.

## Preprocedural Guidance

### Selection of PCI Strategy

**Fig. 3:**
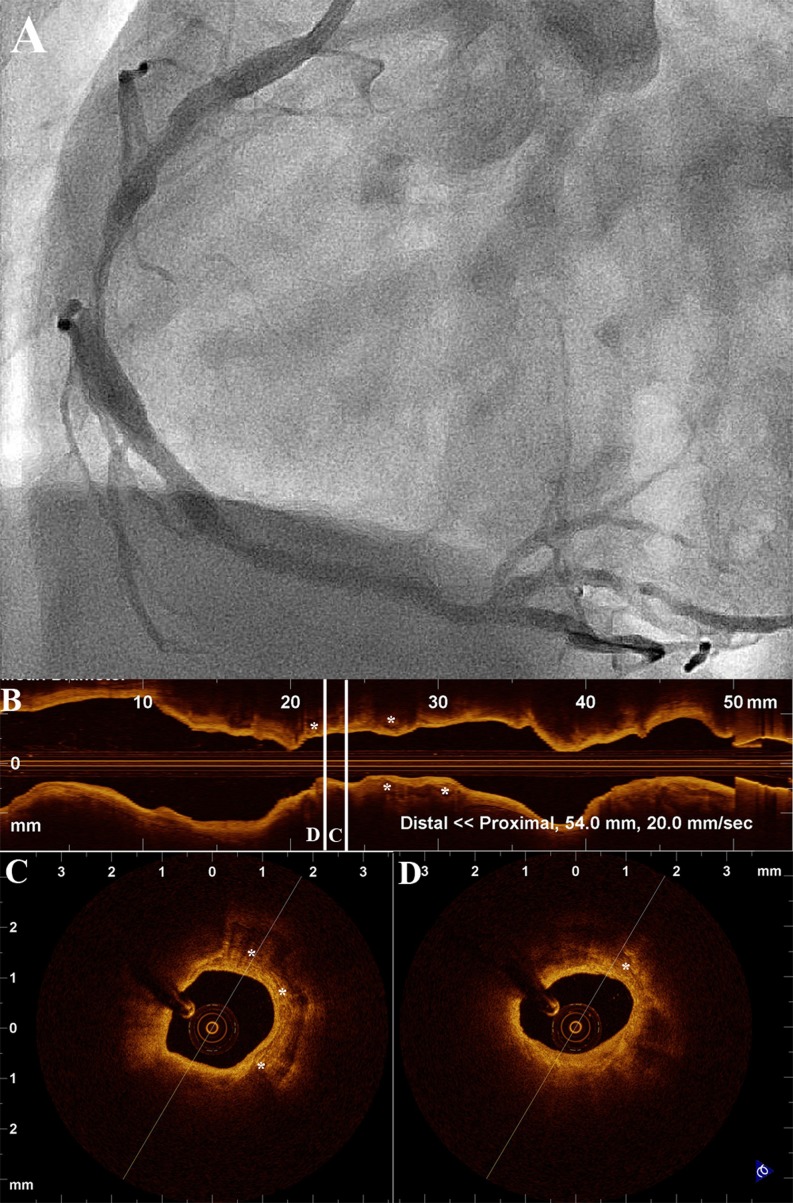
**a** Angiogram of the right coronary artery of a patient with stable angina. **b** L-mode reconstruction of OCT images demonstrating the longitudinal morphology of the lesion. **c, d** Representative OCT cross-sections showing heavily superficially calcified plaque suggesting the need for targeted therapy such as rotablation. *Asterisks* represent calcific depositions

OCT can be used in order to guide decisions about treatment before the procedure. As discussed above, OCT can provide precise area measurements not only of the minimal lumen, but of proximal and distal reference sites as well, and define the lesion length. Therefore, preprocedural measurements by OCT can aid in the proper selection of balloon and stent dimensions. Selection of appropriate balloon size using OCT measurements has been performed in a series of patients undergoing cutting balloon angioplasty for the treatment of in-stent restenosis [35]. Furthermore, OCT has also been used for selection of treatment strategy on the basis of lesion morphology. Given that OCT can quantify calcifications of the target site more accurately than IVUS [36], it could better assess the need for targeted therapies (Fig. 3). In a recent study, pre-interventional OCT assessment of plaque morphology guided the selection of dedicated treatments such as rotablation, thrombectomy, and cutting balloon in one third of patients [37•]. In the same study, decision to defer angioplasty was taken in 18 % of the patients using criteria of IVUS guidance [38], for assessing the severity of the lesion.

**Fig. 4:**
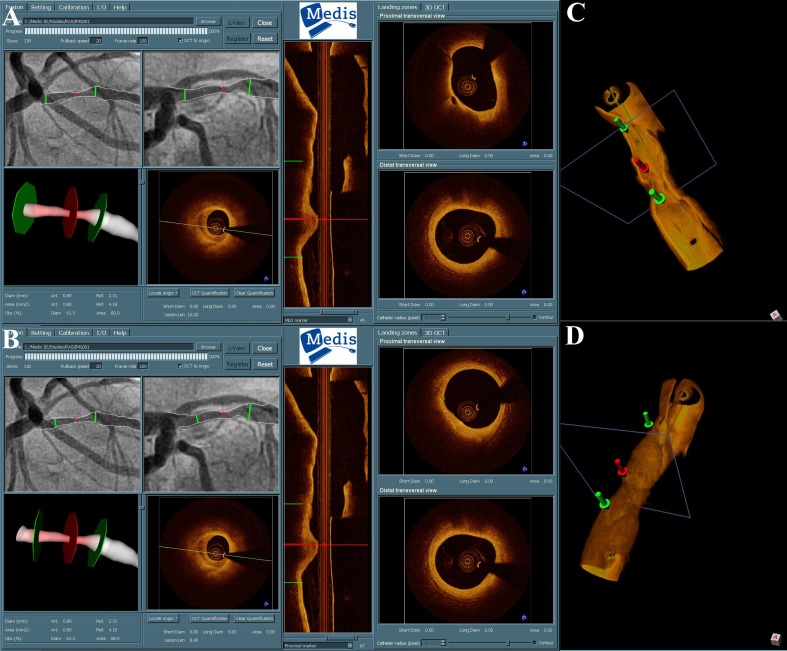
Registration of 3D QCA and OCT using qAngioOCT (Medis Medical Systems BV, Leiden, The Netherlands). **a** Fibrous cap at the minimal lumen area (*red marker*). Images at the right indicate proximal and distal landing zone. In the proximal landing zone there is a thin cap fibroatheroma. **b** If a shorter stent is selected, the proximal landing zone will be at a site with stable plaque. **c, d** Online three-dimensional reconstructions of the segment generated by the software

One of the advantages of OCT guidance is that it can help precisely identify the optimal segment for stent deployment, the so-called “landing zone” (Fig. 4). It has been shown that multiple plaque morphologies may be present in the culprit lesion of a patient with ACS [39]. Moreover, the longitudinal analysis of culprit lesions of ACS has demonstrated that in the majority of cases there is geographic mismatch of the minimal lumen site with the rupture site [40•], indicating the presence of necrotic core plaques in sites remote to the site of the greatest stenosis. Although there is not definitive evidence that stenting should also cover lipid-rich plaques located in shoulder areas, it has been shown that presence of such areas in the edge of the stent is associated with edge dissection, as is the presence of fibrocalcific plaques [41]. Additionally, pathologic studies have shown the association of late stent thrombosis with the presence of struts penetrating areas of necrotic core [42], while as mentioned earlier, possible stent-induced disruption of such areas could lead to impaired vascular healing or raise in cardiac necrosis biomarkers [33, 34].

### Co-registration of Invasive Imaging and Coronary Angiography

While invasive imaging generally offers high spatial resolution, it can be challenging to consolidate the information gained by invasive imaging technologies with the angiogram, and especially, to ensure correct spatial orientation. This is true for all angiographically silent lesions, whereas this phenomenon might be a consequence of vessel overlap, foreshortening or the inability to visualize a complex three-dimensional structure correctly in a two-dimensional image. Often operators use side branches as observed in both imaging modalities as landmarks. This approach is not always the optimal, as side branch ostia are not always clearly visible by angiography and also the assumed calibre of a particular side branch can be underestimated. Currently, a number of technical solutions to this problem are being developed, including the use of pulsed fluoroscopy to track the imaging catheter in real time (Siemens Medical prototype, Erlangen, Germany) [43] or alternatively, approaches for software enabled synchronization of three-dimensional angiography with invasive imaging pullbacks are being developed (Medis Medical Imaging Systems BV, Leiden, The Netherlands) (Fig. 4) [44]. The ultimate goal is an online co-registration of the invasive imaging modality with the coronary angiogram, allowing the operator to scroll through a synchronized dataset. Such information could be useful for planning of the most appropriate interventional strategy and for the longitudinal assessment of atherosclerotic lesions.

### Guidance in Complex Procedures

OCT can also be a tool for guidance during complex procedures. There have been reports of OCT guidance in chronic total occlusions (CTOs) [45]. In such cases, OCT can be used to identify the entry point of the occlusion and to confirm that the guidewire is indeed in the true vessel lumen and has not entered a false lumen. The development of forward-looking OCT catheters will enable the guidance of a CTO crossing by continuous visualization of the position of the guidewire relative to the microchannels.

Bifurcation lesion stenting is another potential application of OCT guidance. By 3-dimensional reconstruction of OCT images it is possible to produce images that indicate the relative position of the main vessel and the side-branch and help select a suitable strategy [46]. Furthermore, three-dimensional models of coronary arteries can help identify the point of re-crossing of a guidewire in a side branch through the cells of a stent implanted in the main vessel, and evaluate possible presence of struts at the ostia of the side branches following kissing balloon dilation [47]. The development of online reconstructions that incorporate three-dimensional angiography and optical coherence tomography images could improve real-time guidance in these subsets of lesions [44, 48•].

## Assessment of Acute Effects of PCI

Pathologic studies have indicated the association of implantation characteristics with late events following stent implantation [49]. Stent strut malapposition has been associated with very late stent thrombosis in IVUS studies [1]. Furthermore, postprocedural lesion assessment has shown that stent expansion, tissue protrusion, and dissection are associated with early stent thrombosis [2, 3], while restenosis is also associated with inadequate stent expansion [3]. Indeed, use of standardized criteria for stent expansion using IVUS guidance and additional treatment based on these criteria was associated with a low restenosis rate in the bare metal stent era [38].

**Fig. 5:**
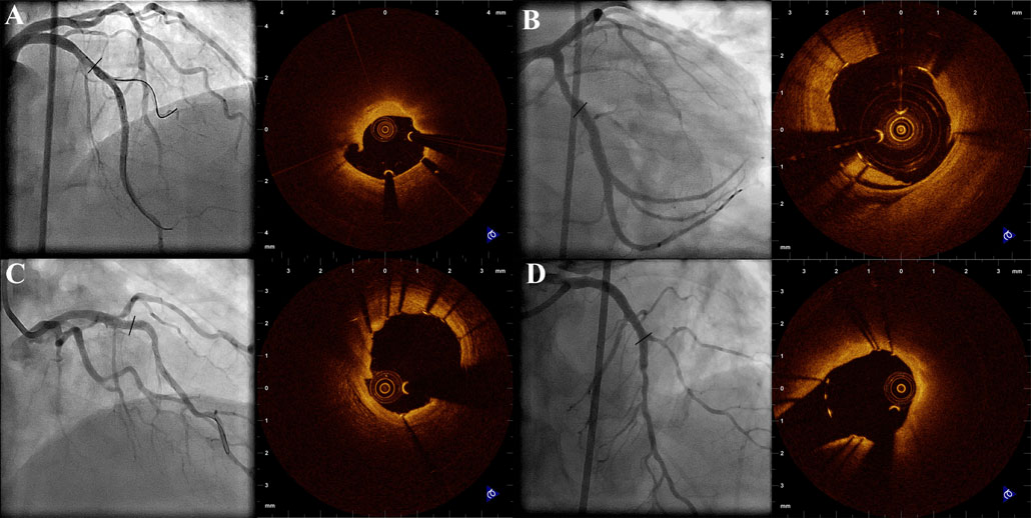
Assessment of acute effects of intervention. **a** Patient with edge dissection after stent implantation at the bifurcation segment. **b** Intra-stent dissection following stent implantation at the left circumflex artery. **c** Tissue protrusion (9 to 10 o’clock) following stent implantation at the middle left anterior descending artery. **d** Stent protrusion to the diagonal branch following primary percutaneous intervention at the left anterior descending artery without kissing balloon dilation

**Fig. 6:**
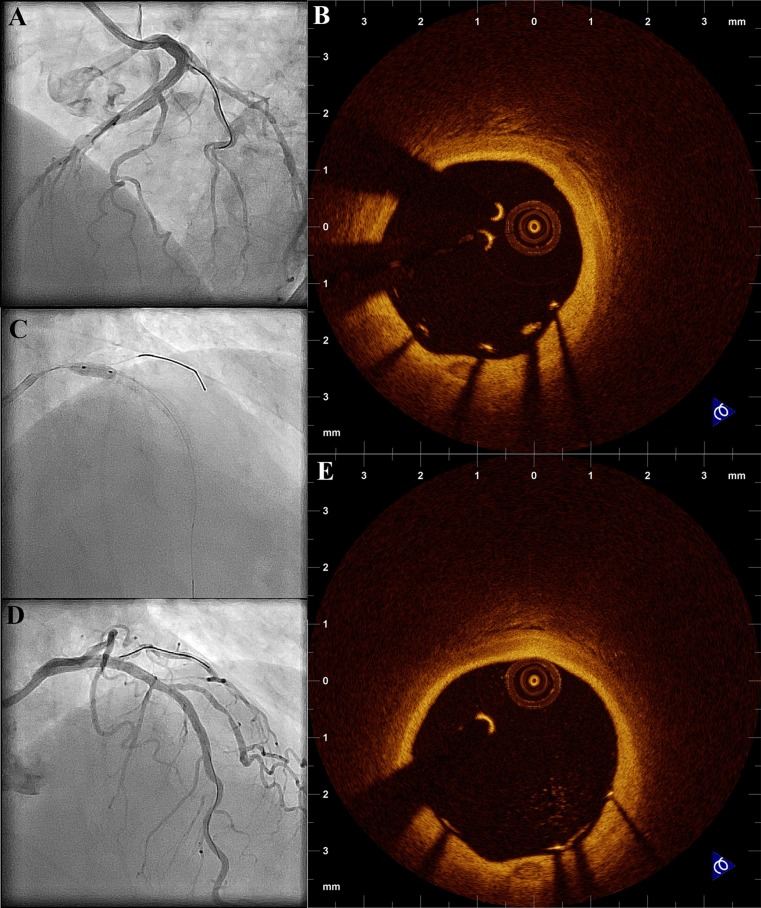
Correction of malapposition at the proximal LAD following stent implantation. **a** Angiographic result following the initial intervention. **b** OCT demonstrating malapposition at the proximal LAD. **c** Post-dilation was performed with a non-compliant balloon. **d** Final angiographic result. **e** OCT shows correction of malapposition (the distance of the strut surface from the vessel wall is lower than the thickness of the strut and polymer, as provided by the manufacturer)

OCT can accurately assess the acute vessel wall response to stent implantation. Due to the high resolution of OCT, tissue prolapse, malapposition, intra-stent thrombus formation, and edge dissection can be detected more accurately than with IVUS (Fig. 5), while stent and luminal measurements by the two modalities are not significantly different [7••, 9, 50]. In a study assessing the effects of stenting on vessel wall morphology, it was shown that the presence of dissections, either at the stent edge or inside the stent, and tissue protrusion was observed in almost all the cases, while in the majority of patients there was some degree of stent malapposition [51•]. Thrombus presence inside the stent was also common, both in stable and in ACS patients [51•]. Furthermore, optical coherence tomography is particularly useful in the assessment of malapposition, due to its superior resolution allowing the detection of small degrees of malapposition not detectable by IVUS with low variability between independent measurements. As OCT signal does not penetrate the surface of stent struts, it is important when calculating the distance of the stent strut from the vessel wall, to subtract the thickness of the strut and polymer from the total distance from strut surface to vessel wall. Acute malapposition has been assessed in a variety of studies, and was found to be associated with plaque and stent characteristics, clinical syndrome, and presence of overlapping stents (Fig. 6) [34, 52].

The significance, however, and prognostic value of the detection of small degrees of malapposition, tissue protrusion, or thrombus that are detectable only with the high-resolution of OCT has not been precisely defined, as with IVUS. Small follow-up studies have tried to assess the impact of these findings with surrogate markers at follow-up [34, 50, 53]. Acute malapposition was more pronounced in implantation compared to follow-up in all of the studies [34, 50, 53]. Conversely, there were cases of initially well-apposed struts that were malapposed at follow-up, possibly due to changes in vessel remodelling [53]. In one of the studies, presence of malapposition was associated with impaired endothelialization and presence of thrombus on struts [53]. Dissections and tissue protrusion, detectable by OCT, tended to resolve at a 6-month follow-up period [50]. Nevertheless, unless larger prospective studies investigate the impact of OCT-detected markers of vessel injury and acute malapposition by OCT on clinical outcome, their role in decision making will remain uncertain.

**Fig. 7:**
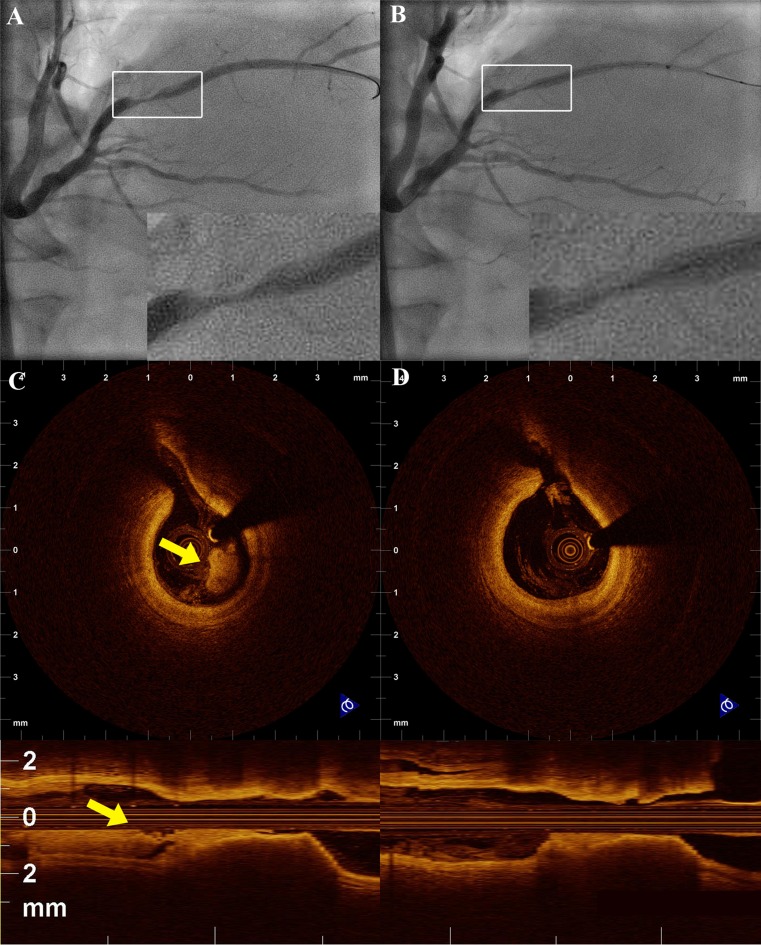
**a** Coronary angiography of the right posterolateral branch of a patient with myocardial infarction. **b** OCT reveals significant thrombus burden (*yellow arrow*). **c** Angiography after manual thrombus aspiration. **d** OCT shows reduction of the thrombus burden, confirming the effectiveness of thrombus aspiration

Post-procedural OCT guidance has not been only used to evaluate vascular injury following stent implantation, but has also been used to assess the effects after PCI with other adjunctive devices such as cutting or scoring balloon and rotational atherectomy [54]. Furthermore, OCT can also be used in ACS in order to assess the role of adjunctive therapies in reducing thrombus burden (Fig. 7) [55, 56].

There is only a handful of studies that have assessed the role of OCT guidance in PCI [12, 35, 37•]. Imola et al. [12] used post-implantation OCT imaging in 74 patients in order to assess the need for further intervention. In 24 patients, additional interventions were performed based on OCT assessment (15 patients with balloon metadilation and 9 patients with additional stent implantation), while in 50 patients the result of the interventions was judged satisfactory, leading to an event-free survival of 98 % at 6-month follow-up. Use of OCT in assessing luminal dimensions after cutting balloon angioplasty has led to the safe upsizing of luminal size in patients without coronary stent implantation [35]. Viceconte et al. [37] report on the outcomes of an OCT-guided strategy for procedural guidance, where in 207 cases, OCT suggested the need of a new stent implantation in 29 and further optimization in 64. No major complications were observed in this study, while serial OCT examinations increased procedural time by a mean of 15.4 ± 8.2 min. Thus, it seems that OCT can be safely used for procedural guidance, but the clinical significance of such an approach remains to be assessed in comparative studies.

## Assessment of Long-Term Outcome After PCI

**Fig. 8:**
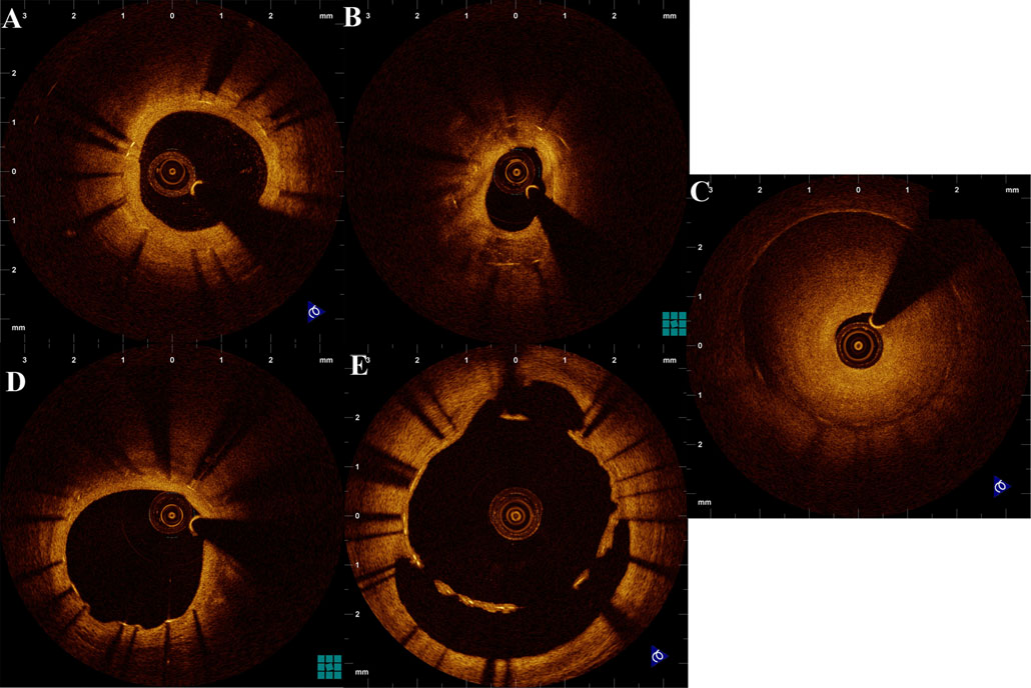
Various patterns of vessel response at the follow-up of stent implantation. **a** Homogeneous tissue coverage without compromise of the lumen. **b** Heterogeneous coverage with areas resembling lipid plaques 3 years after implantation of a drug-eluting stent. **c** Stent restenosis. **d** Presence of uncovered struts in another site of the stent shown in panel B. **e** Stent malapposition at follow-up, with tissue coverage probably corresponding either to fibrin or endothelial coverage

OCT can be a valuable tool for follow-up stent assessment. The association of the extent of stent endothelialization with stent thrombosis in pathologic studies have introduced the need for detailed assessment of stent coverage, as a surrogate marker for stent safety. The superior resolution of OCT allows for near-histology assessment of stent coverage, while the ability of OCT to assess and quantify neointimal hyperplasia with greater accordance with histology compared to IVUS, has clearly been documented (Fig. 8) [57]. Furthermore, the high reproducibility for measurements of neointimal hyperplasia makes OCT a reliable tool for objective evaluation of the degree of restenosis. In view of these advantages, OCT has been used for the assessment of the anti-restenotic efficacy of various devices.

The assessment of neointimal coverage by OCT constitutes the subject of various studies trying to provide insights into subjects related to stent safety. Stent evaluation by OCT can be used to provide detailed information for stent coverage reported to be associated with stent thrombosis in post-mortem studies. It is important to mention that although OCT can detect even minimal tissue coverage at an order of 10 μm, which compares to analysis by optical microscopy, OCT detected coverage could correspond either to true endothelialization or to coverage by fibrin deposits [58]. Therefore, OCT has only about 80 % sensitivity and 95 % specificity for the detection of stent struts lacking endothelial coverage [59]. Nevertheless, due to different optical properties of these components, future developments with characterization based on optical properties [59] may be able to increase the sensitivity for detection of struts without endothelial coverage.

Assessment of tissue coverage has been extensively investigated in the case of the sirolimus-eluting stent, where there are reports from 3 months to 4 years post implantation [60, 61]. All these reports suggest that a small percentage of uncovered struts can be found even 4 years after implantation in sirolimus-eluting stents [61]. Sirolimus-eluting stents, as well as paclitaxel-eluting stents, demonstrate impaired healing compared to bare-metal stents [62, 63]. This does not seem to be the case for the zotarolimus-eluting stent with permanent polymer that is probably the stent with the highest extent of coverage, comparable to that of bare metal stents [64]. Furthermore, comparative studies between sirolimus-eluting and paclitaxel-eluting stents have demonstrated that the latter are associated with an enhanced healing response [65]. A recent OCT substudy of a randomized trial comparing a zotarolimus-stent with biocompatible polymer and the everolimus-eluting stent has shown comparable rates of coverage between these two second-generation stents [66]. Nonetheless, despite the presence of profound differences in vessel healing between different stents, these have not been shown yet to correspond to differences in stent thrombosis, underlining the complexity of mechanisms associated with this entity [67].

Pathologic evidence has shown that bare metal stents are sometimes associated with development of neointimal tissue with characteristics similar to de novo atherosclerosis [68]. Furthermore, recent observations have shown that this entity is also observed in drug-eluting stents at shorter follow-up intervals [69]. The fact that OCT allows for qualitative assessment of restenotic tissue can be used for elucidating the mechanisms behind late stent failure. Various morphological classifications of restenotic tissue have been proposed by OCT [59, 70, 71], however histologic validation was only available in the recent study by Nakano et al. [59], that proposed the use of a classification similar to the classification used for OCT assessment of de novo atheromatosis. According to this study, the differences in the optical properties of the various tissue components can be used for tissue characterization.

Whether such morphological features are implicated in the pathophysiology of very late stent thrombosis is a subject of investigation. Recent data from OCT studies suggest the existence of two discrete mechanisms associated with stent thrombosis: one involving impaired vessel healing after stent implantation with malapposition and incomplete strut coverage, and one associated with rupture of neoatherosclerotic tissue [72]. Histological examination of thrombus aspirates supports this theory by showing differences in the composition of thrombotic debris between these two mechanisms [72, 73]. However, little is known so far about the incidence and predictors of each mechanism, the “vulnerable” neointimal morphological characteristics, and the differences in clinical outcome in these cases. Future OCT studies will provide new insights into the role of each mechanism in the development of very late stent thrombosis and will assess the clinical significance of evaluation of these mechanisms.

## OCT for the Assessment of New Stent Technologies

Due to its superior resolution and the ability to evaluate stent healing and quantify neointimal hyperplasia, OCT is becoming the standard for the assessment of new stent technologies. A new biolimus-eluting stent underwent OCT evaluation in a substudy of the LEADERS trial in order to assess the differences in healing response compared to a sirolimus-eluting stent and was shown to be associated with more complete coverage without increase in neointimal hyperplasia [74]. Moreover, various stents with improvements in release kinetics, coating or matrix design have been evaluated with OCT in order to assess if such improvements correspond to improved healing response as well [75, 76]. Likewise, OCT has been used in a number of studies to evaluate the healing response after treatment with drug-eluting balloons in de novo or in stent restenosis lesions [77].

One of the more fascinating aspects of OCT is the ability to monitor the absorption rate in bioabsorbable stents. OCT has been used for assessment of the biodegradation of the magnesium stent in porcine coronary arteries, as it can demonstrate the reduction in the width of stent struts that is associated with biodegradation [78]. In the case of the bioresorbable everolimus-eluting scaffold (Absorb™, Abbott Vascular, Santa Clara, CA) there have been more extensive animal and human studies [79–81]. Experimental studies have shown that OCT can assess the integration process of the scaffold in long-term observations [81]. In human studies, pilot trials have defined the OCT appearance of various stages of the resorption of the scaffold [79, 80], while OCT has been used for assessment of the vascular healing process and evaluation of differences in resorption and stent strut distribution between the first and the second generation of the scaffold [82], being the best method for measurement of stent length and luminal dimensions [83], as well as assessment of healing in side branches [84].

## Future Research Directions

Intravascular OCT is a powerful and easily applicable tool for the assessment of the coronary artery. Today, OCT image interpretation and analysis is based on a color-coded intensity map. Algorithms enabling automated tissue characterization software will allow for a more objective assessment of plaque morphology [85]. New technologies such as polarization-sensitive OCT will enable the distinction of the collagen content of the coronary plaques [86], while the development of micro-OCT will help the better understanding of the pathophysiological mechanisms of acute coronary syndromes [87].

Despite the numerous clinical uses of OCT, and a wealth of case series and clinical observations illustrating potential use in a clinical setting, no clear indications have been established yet. Future studies, focusing either on unselected population or on specific patient and lesion subsets (i.e., acute coronary syndromes, calcified lesions, bifurcations), will determine the role of OCT in procedural guidance.

Furthermore, given that OCT can provide us with an enormous amount of information about plaque morphology and vessel healing following stent implantation, future research will elucidate which are the critical components of plaque and stent “vulnerability” on which we should focus. Finally, natural history studies investigating the impact of vessel morphology and acute procedural complications, as detected by OCT, on future clinical events need to be implemented. And if an association of such OCT findings with clinical outcome is demonstrated, it remains to be seen if treatment based on those findings can lead to an improvement in outcomes.

## Conclusions

The introduction of OCT has not only provided new insights in the pathophysiology of the plaque and of the vascular response to stenting, but has also offered interventional cardiology a new tool for procedural guidance. Currently, there are no clear indications for OCT in a clinical setting; however, there are numerous fields where useful decision-guiding information could be provided by OCT. These include pre-interventional evaluation of coronary arteries, procedural guidance in coronary interventions, as well as follow-up assessment of vascular healing after stent implantation. The development of three-dimensional models reconstructed from OCT images could perhaps enhance the role of OCT in procedural guidance [44], while automated tissue characterization software will allow for more objective assessment of plaque morphology [85], strengthening the role of OCT as a tool for implementation of natural history studies. Studies aiming to show an association between OCT imaging and improvement in patient-related outcomes will ultimately define the role of OCT in interventional cardiology.
